# Control of Line Tension at Phase-Separated Lipid Domain Boundaries: Monounsaturated Fatty Acids with Different Chain Lengths and Osmotic Pressure

**DOI:** 10.3390/membranes12080781

**Published:** 2022-08-14

**Authors:** Nichaporn Wongsirojkul, Aiko Masuta, Naofumi Shimokawa, Masahiro Takagi

**Affiliations:** School of Materials Science, Japan Advanced Institute of Science and Technology, Nomi City 923-1292, Japan

**Keywords:** lipid membrane, phase separation, monounsaturated fatty acid, osmotic pressure, line tension

## Abstract

Line tension at phase-separated lipid domain boundaries is an important factor that governs the stability of the phase separation. We studied the control of the line tension in lipid membranes composed of dioleoylphosphocholine (DOPC), dipalmitoylphosphocholine (DPPC), and cholesterol (Chol) by the addition of the following three monounsaturated fatty acids (MUFAs) with different chain lengths: palmitoleic acid (PaA), oleic acid (OA), and eicosenoic acid (EiA). In addition, we attempted to alter the line tension by applying osmotic pressure. The phase behavior of the MUFA-containing lipid membranes in the presence and absence of osmotic stress was observed by fluorescence and confocal laser scanning microscopy. The line tension was quantitatively measured from the domain boundary fluctuation by flicker spectroscopy, and the interactions between the lipids and MUFAs were examined by differential scanning calorimetry. PaA and OA, which are shorter MUFAs, decreased the line tension, whereas EiA changed the liquid domain to a solid domain. The osmotic pressure increased the line tension, even in the presence of MUFAs. It may be possible to control the line tension by combining the chemical approach of MUFA addition and the physical approach of applying osmotic pressure.

## 1. Introduction

Biomembranes are primarily composed of amphiphilic phospholipid molecules organized in a bilayer structure. It is believed that the various components of biomembranes, such as phospholipids, cholesterol, and membrane proteins, are not uniformly mixed but rather form spatially heterogeneous raft structures. Because these lipid rafts may be involved in signal transduction and membrane trafficking, elucidation of raft formation is crucial for understanding cellular functions [1,2]. Bilayer membranes and vesicles (spherically closed bilayer membranes), consisting of various types of phospholipids, have been applied as model systems for biomembranes, and compositionally heterogeneous structures have been reported to emerge spontaneously under certain conditions. This phenomenon can be physicochemically interpreted as phase separation, and studies of the phase separation in lipid membranes in relation to raft formation are underway [3].

Lipid membranes composed of saturated phospholipids, unsaturated phospholipids, and cholesterol are commonly used in phase separation studies. In these systems, a liquid-ordered (L_o_) phase rich in saturated lipids and cholesterol and a liquid-disordered (L_d_) phase rich in unsaturated lipids coexist, and the L_o_ phase is regarded as a model of lipid rafts [4,5,6]. The interfacial tension at the phase-separated domain boundary, which is referred to as line tension, is an important parameter when considering the stability of the phase separation, and control over the line tension may allow control over the phase-separated structures. The line tension can be measured from the thermal fluctuation of the phase-separated domain boundary [7,8,9] or by micropipette aspiration [10]. Experimental measurements of the line tension near the critical point [11,12] and in the presence of hybrid lipids [13,14,15,16], vitamin E [17,18], and local anesthetics [19] have been performed.

Unsaturated fatty acids have also been examined from the perspective of line tension control. These compounds have attracted attention, largely owing to their association with health. For example, polyunsaturated fatty acids (PUFAs) present in foods, such as docosahexaenoic acid and eicosapentaenoic acid, may reduce the risk of lifestyle diseases, such as type 2 diabetes mellitus (T2DM) [20] and cardiovascular disease [21]. Moreover, free fatty acids and cholesterol are also related to obesity, and abnormal lipid metabolism, as well as T2DM [22]. In particular, it was suggested that cholesterol catalyzes the aggregation of islet amyloid polypeptides, which are linked to T2DM [23,24]. In addition to PUFAs, monounsaturated fatty acids (MUFAs), such as oleic acid, may exert a beneficial effect on health by increasing the level of high-density lipoprotein cholesterol [25]. Although the effects of unsaturated fatty acids on membrane organization (e.g., phase separation) are not well understood, it is known that the biomembrane composition, which plays a crucial role in membrane organization, is influenced by the diet, including cholesterol and fatty acid intake. Furthermore, the phase behavior of fatty-acid-containing lipid membranes has been observed experimentally, and oleic acid was found to significantly decrease the line tension at the domain boundary [26].

The line tension is also affected by the osmotic pressure. Because a lipid membrane is semipermeable, osmotic pressure is generated upon sandwiching the membrane between solutions of different solute concentrations. Consequently, vesicles shrink or swell depending on the solute concentration gradient across the membrane. In the body, osmotic pressure plays essential roles in metabolism, growth, development, and fluid homeostasis. Furthermore, the membrane lateral tension, resulting from the osmotic pressure, influences the activity of mechanosensitive channels in the membrane [27,28]. For example, in a hypotonic solution, where the external solute concentration is lower than the internal solute concentration, vesicles swell, owing to the water inflow and the resulting membrane lateral tension promotes the formation of phase-separated structures and increases the line tension [29,30,31,32]. The swelling of a vesicle also suppresses its thermal membrane fluctuation and decreases the entropy associated with membrane fluctuation [33]. In general, a homogeneous fluid membrane strongly fluctuates as a result of its flexibility, whereas a phase-separated membrane fluctuates significantly less because of the presence of the rigid ordered phase. The homogeneous membrane is strongly affected by the fluctuation suppression due to swelling and becomes unstable. Therefore, under osmotic stress, the phase-separated structure is relatively stable, the phase separation is promoted, and the line tension increases.

The addition of molecules such as unsaturated fatty acids is a chemical approach to controlling the line tension, whereas the application of osmotic pressure is a physical approach. In this study, we combine these two approaches to achieve control over the line tension. As the unsaturated fatty acids, three MUFAs featuring different chain lengths, namely, palmitoleic acid, oleic acid, and eicosenoic acid, were used. First, the phase separation of the MUFA-containing lipid membranes was observed by fluorescence microscopy. From the domain boundary fluctuation, we quantitatively measured the line tension in the absence and presence of osmotic pressure. The effects of the MUFAs and osmotic pressure on the shape of the vesicles were examined by confocal laser scanning microscopy. In addition, we examined the interaction between lipids and the MUFAs by differential scanning calorimetry (DSC). Finally, we discuss the mechanisms of line tension control by the addition of MUFAs and the application of osmotic pressure.

## 2. Materials and Methods

### 2.1. Materials

The saturated phospholipid 1,2-dipalmitoyl-*sn*-glycero-3-phosphocholine (DPPC), the unsaturated phospholipid 1,2-dioleoyl-*sn*-glycero-3-phosphocholine (DOPC), and cholesterol (Chol) were purchased from Avanti Polar Lipids (Alabaster, AL, USA). Rhodamine B 1,2-dihexadecanoyl-*sn*-glycero-3-phosphoethanolamine (triethylammonium salt; Rho-DHPE) and 1,2-dipalmitoyl-*sn*-glycero-3-phosphoethanolamine-*N*-(7-nitrobenz-2-1,3-benzoxadiazol-4-yl) (ammonium salt; NBD-PE) were obtained from Thermo Fisher Scientific (Waltham, MA, USA) and Avanti Polar Lipids, respectively. Oleic acid (C18:1, *cis*-9; OA), palmitoleic acid (C16:1, *cis*-9; PaA), and d-(+)-glucose were acquired from Nacalai Tesque (Kyoto, Japan). Eicosenoic acid (C20:1, *cis*-9; EiA) was obtained from Larodan Fine Chemicals (Malmö, Sweden). Ultrapure water (specific resistivity ≥ 18 MΩ·cm) was obtained from a Millipore Milli-Q purification system (Burlington, MA, USA). The chemical structures of the lipids and MUFAs used in this study are shown in Figure S1.

### 2.2. Vesicle Preparation

Vesicles were prepared using the natural swelling method. The phospholipids, cholesterol, and MUFAs (0.2 mM) and the fluorescent probes (Rho-DHPE and NBD-PE, 0.1 μM) were dissolved in chloroform to obtain stock solutions, which were then mixed in glass test tubes to afford the desired compositions. Lipid films were prepared by evaporating the organic solvent under a flow of nitrogen gas, then dried under vacuum for 3 h. The films were then pre-heated using 5 µL of Milli-Q water for 10 min at 55 °C and hydrated using 200 mM glucose solution for 3 h at 37 °C.

In the osmotic pressure experiment, swollen vesicles were obtained by mixing the vesicle solution with Milli-Q water in a ratio of 1:9. This dilution step reduced the glucose concentration outside of the vesicles from 200 to 20 mM, such that the difference in glucose concentration between the inside and outside of the vesicles was Δ*C* = *C*_i_ − *C*_o_ = 180 mM, where *C*_i_ and *C*_o_ denote the glucose concentrations inside and outside of the vesicles, respectively. Consequently, water influx occurred, owing to the concentration gradient across the lipid membrane.

### 2.3. Microscopy

The vesicle solutions were placed on a glass slide and covered with a smaller glass coverslip, with a spacing of ca. 0.1 mm. The vesicle solutions were observed at room temperature under a fluorescence microscope (IX71, Olympus, Tokyo, Japan) and a confocal laser scanning microscope (FV1000, Olympus, Tokyo, Japan). Rho-DHPE and NBD-PE were used as fluorescent dyes. The fluorescence of Rho-DHPE was monitored using a standard U-MWIG filter set, with an excitation wavelength (λ_ex_) of 530–550 nm and an emission wavelength (λ_em_) of 575 nm. The fluorescence of NBD-PE was monitored using a U-MNIBA filter, with a λ_ex_ of 470–495 nm and a λ_em_ of 510–550 nm. The observation time for each slide was restricted to 90 s to avoid the occurrence of photo-oxidation.

### 2.4. Flicker Spectroscopy of Domain Boundary Fluctuation

The line tension (γ) at a fluctuating domain boundary was determined by analyzing a movie of a phase-separated fluctuating domain (100×, objective lens) recorded over > 3 s (>90 frames, 30 frames/s). The pixel size of the obtained microscopic images was 100 nm × 100 nm. By binarizing the obtained movie with ImageJ, the radial fluctuation of the domain was identified. The domain radius (*r*) was plotted as a function of the polar angle (ψ) [7,8,9] and expressed in terms of a Fourier series expansion, as shown by the following equation:

(1) $$r\left(\psi \right)=r_{\mathrm{av}}\left[1+a_{o}+\sum_{k=1}^{\infty }a_{k}\cos\left(k\psi \right)+\sum_{k=1}^{\infty }b_{k}\sin\left(k\psi \right)\;\right]$$

where $r_{\mathrm{av}}$ is the average domain radius, k is the mode number, and $a_{k}\;$ and $b_{k}$ are the Fourier coefficients. The relationship between the fluctuation and the excess free energy can be written as

(2) $$\Delta F≃\frac{\pi r_{\mathrm{av}}}{2}\gamma \sum_{k=2}^{\infty }\left(k^{2}-1\right)\left(a_{k}^{2}+b_{k}^{2}\right)$$

where γ is the line tension. The free energy for each independent mode is $k_{B}T$ from the generalized equipartition theorem, where $k_{B}$ is the Boltzmann constant and T is the absolute temperature. Therefore, we can obtain

(3) $$\langle a_{k}^{2}\rangle +\langle b_{k}^{2}\rangle =\frac{2k_{B}T}{\pi r_{\mathrm{av}}\gamma }\left(\frac{1}{k^{2}-1}\right)$$

where 〈…〉 is the average value for all images. The line tension γ can be obtained by fitting the experimental data with Equation (3). Five to ten fluctuating domains were analyzed for each composition and condition. The details of the analysis are provided in Figure S2.

### 2.5. Differential Scanning Calorimetry

DPPC, cholesterol, and the MUFAs were dissolved in chloroform at a concentration of 300 mM. Lipid films were then prepared by mixing these components in the desired ratio with a total volume of 30 μL. The chloroform was removed under nitrogen gas flow and the samples were dried in a vacuum desiccator for 3 h. For the hydration step, 60 μL of Milli-Q water was deposited onto the film to obtain a final concentration of 150 mM. The solutions were mixed and homogenized using a vortex mixer and sonicated at 60 °C for 1 h to produce the vesicles from the bottom of the tube. All of the thermographs were recorded on a DSC822 system (Mettler Toledo, Switzerland) from 20 to 60 °C, with a heating/cooling rate of 5 °C/min. Approximately 12 μL of the homogenized sample was placed into the aluminum sample pan. The same weight of Milli-Q water was used in the reference cell. Each sample was measured over three cycles of heating and cooling and each measurement was repeated at least three times to ensure the reproducibility of the results.

The obtained asymmetric thermographs were assumed to be described by a linear combination of two independent transitions. The thermographs were deconvolved by performing Gaussian two-peak fitting analysis in OriginPro 2018.

## 3. Results

### 3.1. Phase Behavior of MUFA-Containing Lipid Membranes

**Figure 1:**
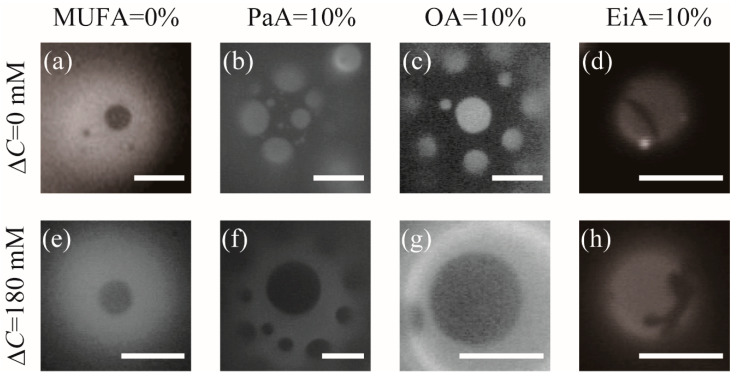
Phase separation observed by fluorescence microscopy for DOPC/DPPC/MUFA/Chol: (**a**,**e**) 40/40/0/20, (**b**,**f**) 30/40/10/20 (MUFA = PaA), (**c**,**g**) 30/40/10/20 (MUFA = OA), and (**d**,**h**) 30/40/10/20 (MUFA = EiA). The upper (**a**–**d**) and lower (**e**–**h**) rows are the images at Δ*C* = 0 (without osmotic stress) and 180 mM (with osmotic stress), respectively. Scale bars are 5 μm.

To examine the effects of MUFAs on the phase separation of lipid membranes, the phase behavior of the DOPC/DPPC/Chol = 40/40/20 system without any MUFAs was first observed by fluorescence microscopy at room temperature (24 ± 2 °C) using Rho-DHPE, as shown in Figure 1a. Rho-DHPE was incorporated into the DOPC-rich phase (L_d_ phase), causing a brightened region, while the DPPC- and Chol-rich region (L_o_ phase) remained dark. To understand the effects of incorporating MUFAs in lipid membranes, we partially replaced the unsaturated component DOPC with three MUFAs of different chain lengths, namely palmitoleic acid (C16:1, *cis*-9; PaA), oleic acid (C18:1, *cis*-9; OA), and eicosenoic acid (C20:1, *cis*-9; EiA). Figure 1b–d show the phase behavior of the lipid membranes incorporated with PaA, OA, and EiA (10%), respectively. In a previous study, the phase separation was investigated when DOPC was replaced with various amounts of OA and it was reported that phase separation could be observed at OA contents of 10–30%, whereas no phase-separated structures were identified when all of the DOPC was replaced with OA (40%) [26]. In addition, domain boundary fluctuation, which indicates the reduction in line tension, was observed in the OA-containing lipid membranes. Our observations were in agreement with this previous study. As shown in Figure 1b,c, the domain shape was not circular and the domain boundary fluctuated continuously. Therefore, these domains are liquid domains. Interestingly, in the case of EiA, the domain boundary fluctuation was not observed. Rather, the formation of vesicles was suppressed, the vesicle size became smaller, and the phase separation between the solid-ordered (S_o_) phase and L_d_ phase mainly occurred, as shown in Figure 1d. In this case, the brightened region is the DOPC-rich L_d_ phase, and the dark region is the DPPC-rich S_o_ phase.

Next, we investigated the influence of osmotic stress on the phase behavior of the MUFA-containing phase-separated vesicles by adding Milli-Q water to vesicle solutions hydrated with 200 mM glucose solution. Because the added water diluted only the solution outside of the vesicles, this resulted in a difference in glucose concentration on the two sides of the membrane. A solution with a lower solute concentration than that inside the vesicles is referred to as a hypotonic solution. Because the lipid membrane is semipermeable, this applies an osmotic pressure and induces water inflow through the membrane. As a result, the vesicles become swollen and the membrane lateral tension increases. This osmotic-pressure-induced lateral tension increases the line tension at the phase-separated domain boundary [30]. Therefore, we observed the phase behavior of the MUFA-containing phase-separated vesicles, while monitoring the domain boundary fluctuation in the hypotonic solution (Δ*C* = 180 mM).

The fluorescence microscopy image of the DOPC/DPPC/Chol = 40/40/20 system without any MUFAs at Δ*C* = 180 mM is shown in Figure 1e. There is no change in the domain shape and it is a liquid domain. The domain boundary did not fluctuate, as in the case of Δ*C* = 0 mM (Figure 1a). Figure 1f–h show the fluorescence microscopy images of the MUFA-containing lipid membranes under osmotic stress (Δ*C* = 180 mM). In the case of PaA and OA, the domain shape was slightly distorted from the circle, indicating that the domain boundary fluctuates Δ*C* = 0 mM (Figure 1b,c). Upon applying the osmotic pressure, however, the domain shape became almost circular, as shown in Figure 1f,g, indicating that the domain boundary fluctuation was suppressed. These circular domains can be considered liquid domains. For the EiA-containing membranes, we did not observe any significant effects of the osmotic pressure on the phase behavior, and S_o_ domain formation was observed, as in the case of Δ*C* = 0 mM (Figure 1h).

### 3.2. Line Tension of MUFA-Containing Phase-Separated Lipid Membranes

The line tension (γ) at a domain boundary can provide important insights into the stability of phase separation. Therefore, we studied the effects of the various MUFAs on the line tension based on the flicker spectroscopy measurements of the domain boundary fluctuation. We excluded EiA from these measurements because the incorporation of this MUFA led to S_o_/L_d_ phase separation.

The fluctuating domain under each condition was recorded for more than 3 s (30 frames/s) at room temperature for 5–10 different vesicles. The radii of the isolated domains at the centers of the vesicles were traced and analyzed using the ImageJ software. Vesicles with crowded domains were not used for the line tension measurements to eliminate any effects of the surrounding domains on the domain fluctuation. The line tension values were calculated using the slope of a plot of $\langle a_{k}^{2}\rangle +\langle b_{k}^{2}\rangle$ (or $r_{\mathrm{av}}\left[\langle a_{k}^{2}\rangle +\langle b_{k}^{2}\rangle \right]$) versus $1/\left(k^{2}+1\right)$ by Equation (3). The details of the line tension analysis can be found in Figure S2.

**Figure 2:**
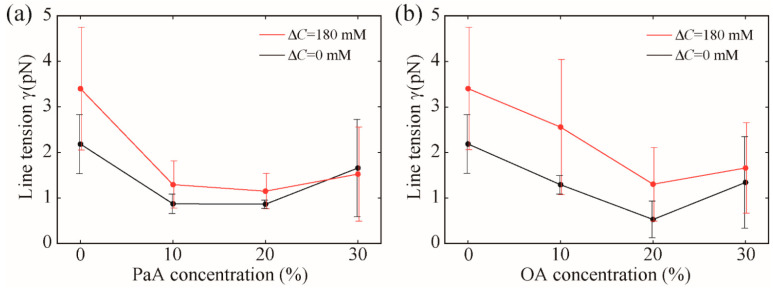
Line tension at the L_o_/L_d_ phase boundary for (**a**) DOPC/DPPC/PaA/Chol and (**b**) DOPC/DPPC/OA/Chol. PaA (or OA) = 0%, 10%, 20%, and 30% correspond to DOPC/DPPC/PaA(OA)/Chol = 40/40/0/20, 30/40/10/20, 20/40/20/20, and 10/40/30/20, respectively. The black and red lines represent the results for Δ*C* = 0 mM (without osmotic stress) and 180 mM (with osmotic stress), respectively.

Our measured line tension value at room temperature for the phase-separated domain in the DOPC/DPPC/Chol = 40/40/20 system without any MUFAs was 2.18 ± 0.65 pN. This value is consistent with those reported in previous works (γ ≈ 3 pN) [10,19,26,30]. Next, we measured the line tension for the lipid membranes containing PaA and OA, and the results are represented by the black lines in Figure 2. The exact values obtained from these measurements were 0.87 ± 0.21 pN (PaA = 10%), 0.86 ± 0.09 pN (PaA = 20%), 1.66 ± 1.07 pN (PaA = 30%), 1.29 ± 0.21 pN (OA = 10%), 0.53 ± 0.40 pN (OA = 20%), and 1.34 ± 1.00 pN (OA = 30%). Thus, except for the OA and PaA contents of 30%, the line tension decreased with increasing OA and PaA concentration, which is in accordance with the results of the previous study using OA [26]. The line tension values for PaA contents of 10% and 20% were almost the same and the line tension was considerably reduced at a PaA content of 10%. In contrast, the line tension increased at PaA and OA contents of 30%. This may be attributable to the inhibition of stable vesicle formation and large deviations in the lipid composition between vesicles at high fatty acid concentrations.

Next, we measured the line tension under osmotic stress, as represented by the red lines in Figure 2. The exact values obtained from these measurements at Δ*C* = 180 mM were 3.36 ± 1.35 pN (MUFA = 0%), 1.30 ± 0.52 pN (PaA = 10%), 1.15 ± 0.39 pN (PaA = 20%), 1.53 ± 1.03 pN (PaA = 30%), 2.56 ± 1.48 pN (OA = 10%), 1.30 ± 0.81 pN (OA = 20%), and 1.66 ± 0.99 pN (OA = 30%). Therefore, the line tension increased upon the application of osmotic stress under all conditions. This finding is consistent with the domain observations shown in Figure 1. Thus, it was revealed that the line tension increased, owing to the osmotic pressure, even in the presence of MUFAs.

One must note that flicker spectroscopy has been reported to be suitable for line tension measurements below 1 pN [8]. In particular, when the osmotic pressure is applied, the line tension becomes large and is out of the measurement range. Although the measurements under the osmotic stress lack quantification, there is no difference in the essential result that the line tension increases due to the osmotic pressure. In the future, we will try to produce quantitative estimates using the other methods, such as micropipette aspiration.

### 3.3. Shape of MUFA-Containing Vesicles

**Figure 3:**
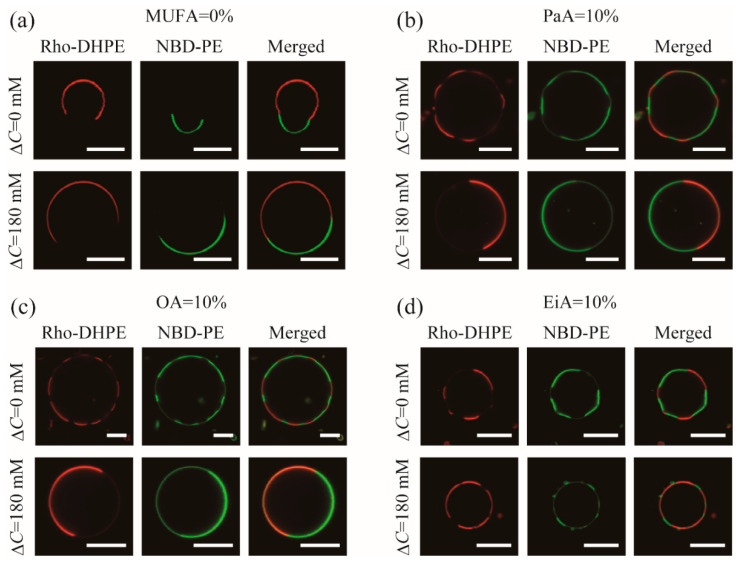
Phase-separated vesicles observed by confocal laser scanning microscopy for (**a**) DOPC/DPPC/MUFA/Chol = 40/40/0/20, (**b**) 30/40/10/20 (MUFA = PaA), (**c**) 30/40/10/20 (MUFA = OA), and (**d**) 30/40/10/20 (MUFA = EiA) at Δ*C* = 0 (without osmotic stress) and 180 mM (with osmotic stress). The red and green colors correspond to Rho-DHPE and NBD-PE, respectively. Scale bars are 10 μm.

The shapes of the MUFA-containing vesicles in the presence and absence of osmotic pressure were observed by confocal laser scanning microscopy, as shown in Figure 3. Rho-DHPE and NBD-PE were used as fluorescent probes for the DOPC-rich and DPPC-rich phases, respectively. Figure 3a shows the vesicle shape without any MUFAs. In the absence of osmotic stress, budding of the liquid domain was observed, as shown in the upper row of Figure 3a. On the other hand, in the presence of osmotic stress, such non-spherical vesicles could not be found, owing to the increased inner pressure of the vesicles, as shown in the lower row of Figure 3a. The vesicles containing PaA or OA displayed polyhedral shapes in the absence of osmotic stress (Figure 3b,c). In particular, the L_o_ domains indicated by the green color became flat and several domains were observed. This tendency is in agreement with the results observed for OA-containing vesicles in the previous study [26]. As will be discussed later, such polyhedral vesicles appear as a result of the reduced line tension. In the presence of osmotic stress, the vesicle shape became spherical, as shown in Figure 3b,c. In addition, the domain size increased while the number of domains decreased, which implies an increase in the line tension. As shown in Figure 3d, we also observed polyhedral vesicles for EiA. As will be discussed later, EiA, which promotes S_o_ domain formation, affords polyhedral vesicles through a distinct mechanism that is independent of line tension reduction. When osmotic pressure was applied, spherical vesicles were observed as for the other MUFAs.

### 3.4. DSC Measurements of MUFA-Containing Membranes

**Figure 4:**
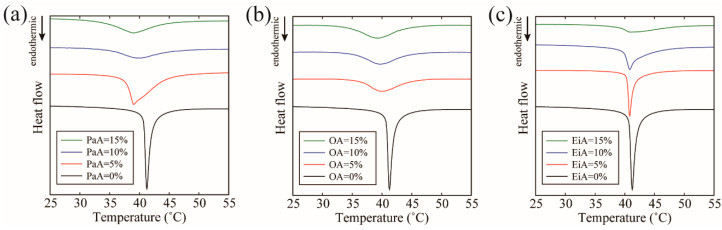
DSC thermographs for (**a**) DPPC/PaA/Chol, (**b**) DPPC/OA/Chol, and (**c**) DPPC/EiA/Chol. The black, red, blue, and green lines indicate DPPC/MUFA/Chol ratios of 90/0/10, 85/5/10, 80/10/10, and 75/15/10, respectively.

To investigate the interactions between the MUFAs and lipids, DSC measurements were performed. The obtained thermographs are presented in Figure 4. For the DPPC/Chol = 90/10 system without any MUFAs, we obtained an asymmetric thermograph with a peak at 41 °C, as shown by the black lines in Figure 4a–c. For both PaA and OA, this peak shifted toward lower temperature with increasing MUFA concentration, as shown in Figure 4a,b. In addition, at PaA or OA contents of 10% and 15%, the obtained thermographs were almost symmetrical. In contrast, for EiA, the peak position did not change significantly, even as the EiA concentration was increased. In addition, the peaks retained an asymmetric shape.

**Figure 5:**
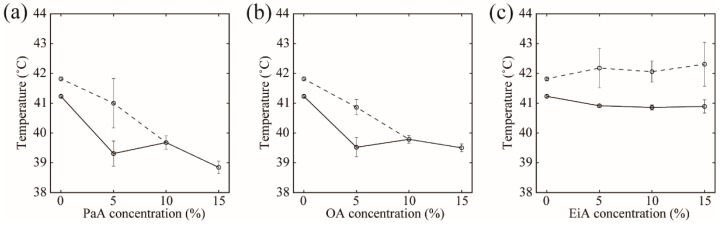
Variation in the peak temperatures obtained from peak deconvolution for (**a**) DPPC/PaA/Chol, (**b**) DPPC/OA/Chol, and (**c**) DPPC/EiA/Chol. MUFA contents of 0%, 5%, 10%, and 15% correspond to DPPC/MUFA/Chol ratios of 90/0/10, 85/5/10, 80/10/10, and 75/15/10, respectively. The solid and dashed lines indicate the lower- and higher-temperature peaks, respectively. Because we obtained symmetric thermographs at PaA and OA contents of 10% and 15%, we have simply plotted the peak temperatures from the thermographs in these cases.

We considered that it may be possible to express the asymmetric thermographs obtained for some compositions as the sum of two Gaussian functions. The results of peak deconvolution can be found in Figure S3. For the DPPC/Chol = 90/10 system, we identified two peaks located at 41.3 and 41.8 °C, which is consistent with the previous report [34]. Peak deconvolution was performed for the MUFA-containing systems in the same manner, and the changes in the two peak temperatures are plotted in Figure 5, where the solid and dashed lines correspond to the positions of the peaks at lower and higher temperatures, respectively. Because we obtained symmetric thermographs at PaA and OA contents of 10% and 15%, we simply plotted the experimentally observed peak temperature in these cases. In general, the lower-temperature peak was sharp and corresponded to the Chol-poor phase, whereas the higher-temperature peak was broad and corresponded to the Chol-rich phase in our measurement range (see Figure S3) [34].

For PaA and OA, lower- and higher-temperature peaks decreased as MUFA content increased, as shown in Figure 5a,b. This implied that PaA and OA interact with DPPC unfavorably. On the other hand, for EiA, both peak temperatures did not change much when the amount of EiA was increased, as shown in Figure 5c. We will discuss this point in the Discussion section.

## 4. Discussion

We found that the addition of PaA or OA decreased the line tension. Because PaA and OA are MUFAs, they were mainly partitioned into the DOPC-rich L_d_ phase. However, a limited amount of PaA or OA, which are smaller than phospholipids, could also be incorporated into the DPPC-rich L_o_ phase. As demonstrated by the DSC results, PaA and OA interacted with DPPC unfavorably. The small amount of PaA or OA slightly disturbed the DPPC chain ordering, and the physical property differences between the L_o_ and L_d_ phases (e.g., chain ordering, membrane thickness, and spontaneous curvature) became smaller. As a result, the line tension decreased upon the addition of PaA or OA.

We observed S_o_/L_d_ phase separation in the EiA-containing lipid membranes. Moreover, in the DSC measurements, the lower- and higher-temperature peaks did not change very much as the EiA content increased. The formation of the S_o_ phase means that the amount of Chol in the DPPC-rich phase has decreased. This suggests that EiA interacts more favorably with DPPC than Chol. Since EiA has a double bond, EiA may disturb the chain ordering of DPPC. On the other hand, EiA has a longer hydrophobic chain than PaA and OA, and it is expected to have a sufficiently strong hydrophobic interaction between DPPC and EiA. Therefore, EiA interacted more favorably with DPPC than Chol, and less Chol interacted with DPPC. As a result, the S_o_ phase formation was induced. To make this point clear, it will be important to investigate the localization of EiA and Chol in the phase-separated membranes by using specific fluorescent probes and spectroscopy. The experimental results were summarized in a schematic illustration in Figure S4.

The application of osmotic pressure suppressed the membrane fluctuation. Therefore, the osmotic stress promoted the formation of a rigid phase with relatively small membrane fluctuation [29]. PaA and OA disturb the chain ordering of the L_o_ phase, resulting in large membrane fluctuation. Such behavior was not favored in the presence of osmotic stress. Thus, the amount of PaA or OA in the L_o_ phase decreased to prevent the membrane from undergoing large fluctuations, and the line tension reduction due to PaA or OA addition became smaller upon the application of osmotic stress. In the case of EiA, the rigid S_o_ phase was formed. Because this is the same trend as the stabilization induced by the application of osmotic pressure, it is likely that no osmotic-pressure-induced changes were observed. In the present study, we considered the localization of MUFAs in phase-separated membranes based on the results of fluorescence microscopy and DSC measurements. In the future, it will be important to investigate the localization directly using MUFA-specific fluorescent probes and infrared spectroscopy.

In addition, we have discussed the effects of the osmotic pressure mainly based on the suppression of the membrane fluctuation. Phase separation is usually interpreted in terms of the lipid interactions and the mixing entropy. The suppression of the membrane fluctuation is the suppression of the vertical movement of the membrane, while the mixing entropy and interactions within membranes are lateral. Therefore, we believe that the changes in the interactions and mixing entropy due to the osmotic stress are limited. However, it will be important to consider what extent they actually change with the osmotic pressure, both experimentally and theoretically.

It is interesting that the phase behavior for PaA and OA was markedly different from that for EiA, despite only small structural differences between the three MUFAs. MUFAs disturb the chain ordering of DPPC, owing to the presence of the double bonds, although the greater chain length of EiA may also lead to strong hydrophobic interactions. PaA and OA have smaller chain lengths, and the disruptive influence of the unsaturated chains presumably outweighed the hydrophobic attraction. In other words, in *cis*-9 MUFAs, the phase behavior of lipid membranes (DOPC/DPPC/Chol) appears to change significantly between C18 (OA) and C20 (EiA). In the future, it would be valuable to systematically investigate the phase behavior for MUFAs with different double bond positions.

For PaA and OA, the vesicles with liquid domains became polyhedral when the line tension decreased. In the case of EiA, the vesicles with solid domains became polyhedral, even though the line tension did not decrease. These deformation mechanisms have been explained by a theoretical model [26]. When the vesicle shape is described by the curvature energy, line energy at the phase-separated domain boundary, and membrane surface energy, the domains should be flat to decrease the curvature energy. However, the formation of one flat domain greatly deforms the vesicle shape and increases the membrane surface energy. Therefore, the energy is reduced by increasing the number of domains and making the vesicle more spherical. Although the line energy increases upon increasing the number of domains, it does not increase markedly for a small line tension. Consequently, a polyhedral vesicle with many flat domains is formed. Next, although EiA did not reduce the line tension, we also observed polyhedral vesicles for the EiA-containing membranes. This occurred because EiA transforms the L_o_ phase into the S_o_ phase, with a dramatic increase in the bending rigidity. To decrease the curvature energy, the domains become flat. This behavior, resulting in rigid and flattened domains, was also observed for a saturated fatty acid and a *trans* fatty acid [26], with which EiA displayed similar behavior. Upon the application of osmotic pressure, these polyhedral vesicles disappeared, owing to the large energetic penalty associated with a non-spherical shape in the case of high internal pressure.

## 5. Conclusions

We have examined the phase behavior of MUFA-containing lipid membranes and measured the line tension at the phase-separated domain boundary by flicker spectroscopy. PaA and OA significantly decreased the line tension, resulting in domain boundary fluctuation. Moreover, the line tension increased in the presence of osmotic stress for both MUFA systems. On the other hand, EiA did not reduce the line tension, but rather transformed the L_o_ phase into the S_o_ phase. Because of the line tension reduction for PaA and OA and the domain rigidification for EiA, the vesicle shape became polyhedral. The obtained results were supported by the DSC measurements.

The line tension is an important factor that governs the stability of phase separation. Under isothermal conditions, we found that the line tension can be decreased or increased by the addition of MUFAs or the application of osmotic stress. By combining these two approaches, it may be possible to precisely control the line tension without changing the temperature.

## Data Availability

Data is contained within the article or Supplementary Material. The data presented in this study are available in the main text and the supplementary materials.

## Supplementary Materials

The following are available online at https://www.mdpi.com/article/10.3390/membranes12080781/s1, Figure S1: Chemical structures of lipids and MUFAs; Figure S2: Analysis procedure for line tension measurement; Figure S3: Peak deconvolution of DSC thermographs; Figure S4: Schematic illustration summarizing the experimental results.
