# Supplementary material for: Control of Line Tension at Phase-Separated Lipid Domain Boundaries: Monounsaturated Fatty Acids with Different Chain Lengths and Osmotic Pressure
Source: Membranes (Basel). 2022 Aug 14;12(8):781. doi: 10.3390/membranes12080781 (PMC9415386; doi:10.3390/membranes12080781)
Supplement: Supplementary file 1 [file membranes-12-00781-s001.zip › membranes-1810243-supplementary.pdf]

membranes

Supplementary Materials

Control of Line Tension at Phase-Separated Lipid Domain Boundaries: Monounsaturated Fatty Acids with Different Chain Lengths and Osmotic Pressure

Nichaporn Wongsirojkul, Aiko Masuta, Naofumi Shimokawa *, and Masahiro Takagi *

School of Materials Science, Japan Advanced Institute of Science and Technology, Nomi City 923-1292, Japan
* Correspondence: nshimo@jaist.ac.jp (N.S.); takagi@jaist.ac.jp (M.T.); Tel.: +81-761-51-1650

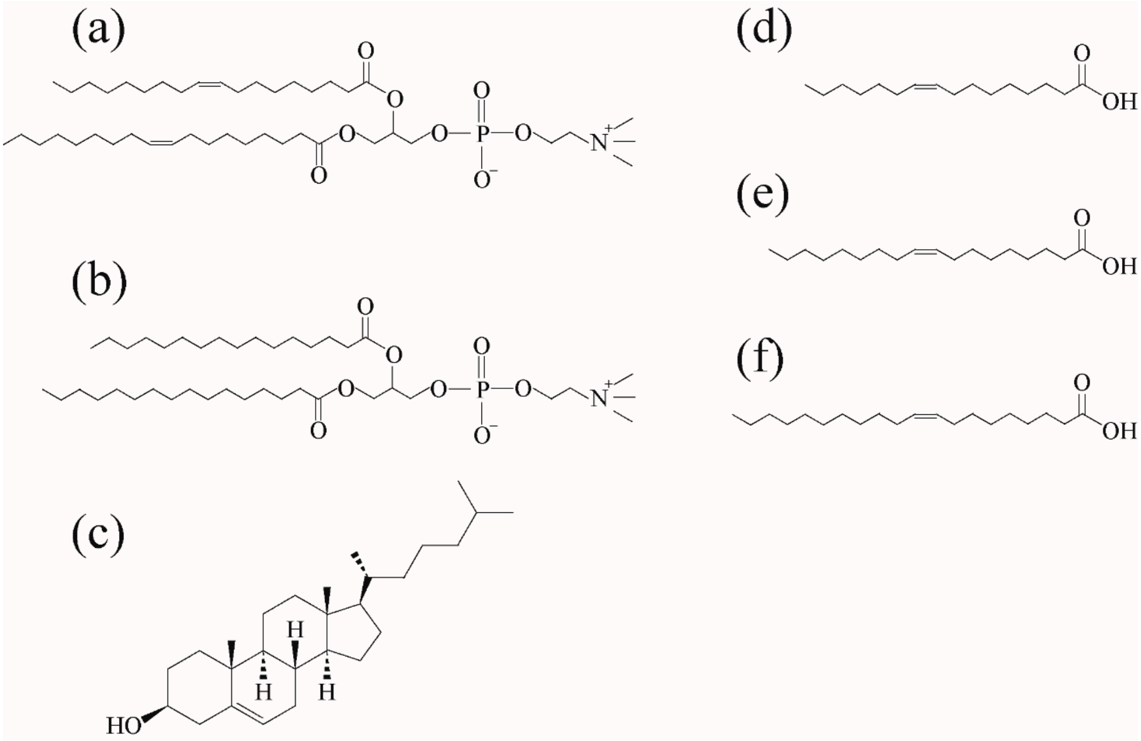

Figure S1. Chemical structures of lipids and monounsaturated fatty acids (MUFAs): (**a**) 1,2-dioleoyl-*sn*-glycero-3-phosphocholine (DOPC), (**b**) 1,2-dipalmitoyl-*sn*-glycero-3-phosphocholine (DPPC), (**c**) cholesterol (Chol), (**d**) palmitoleic acid (C16:1, *cis*-9; PaA), (**e**) oleic acid (C18:1, *cis*-9; OA), and (**f**) eicosenoic acid (C20:1, *cis*-9; EiA).

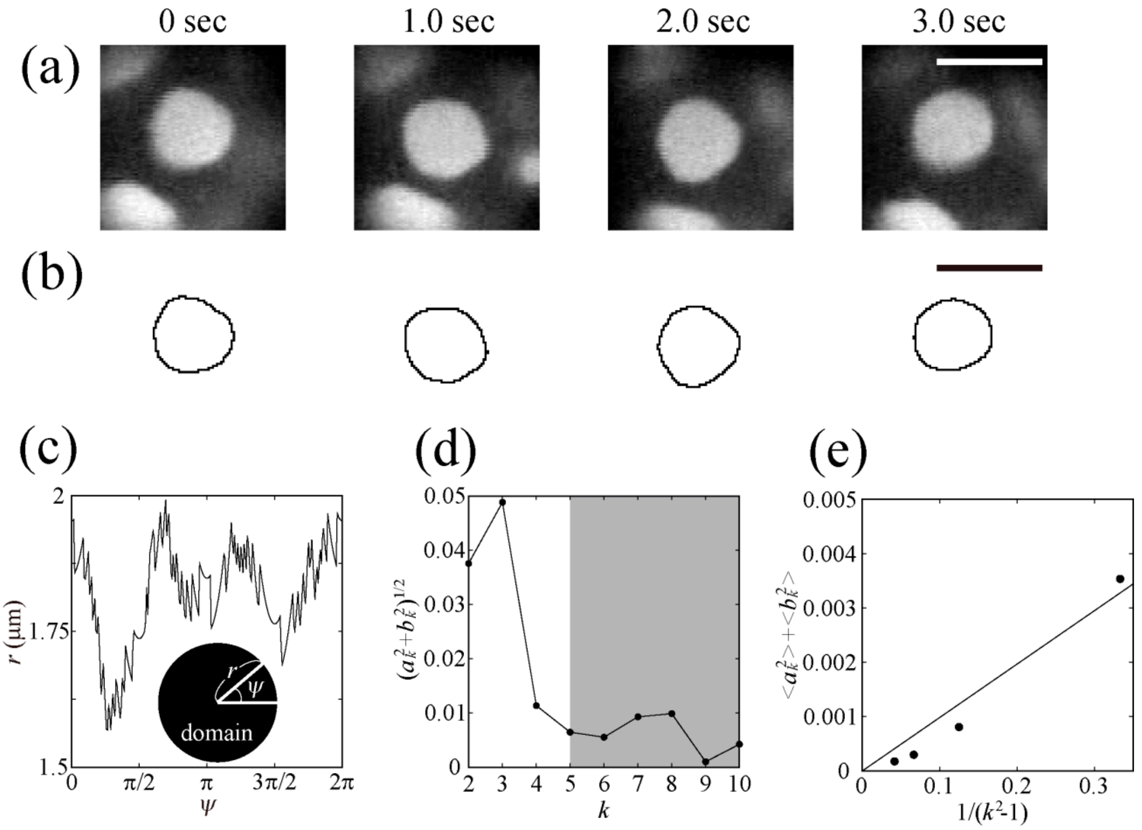

Figure S2. (**a**) Example of fluctuating domain boundaries observed by fluorescence microscopy and (**b**) corresponding domain outlines (DOPC/DPPC/Chol/OA=20/40/20/20 and ΔC = 0 mM). Scale bars are 5 μm. (**c**) Fluctuation of domain radius (r) as a function of polar angle (ψ for time = 0 s). (**d**) Power spectrum calculated from (**c**) using Equation (1) in the main text. (**e**) Average Fourier coefficients obtained from all images plotted against $1/(k^2–1)$ and the fitting line. The obtained waves with k > 5 exceed the resolution and are not detected correctly in our experiment (the shaded region in (**d**)), and the power spectrum also fluctuates randomly in this region. Therefore, we calculated the line tension using only k = 2, 3, 4, and 5.

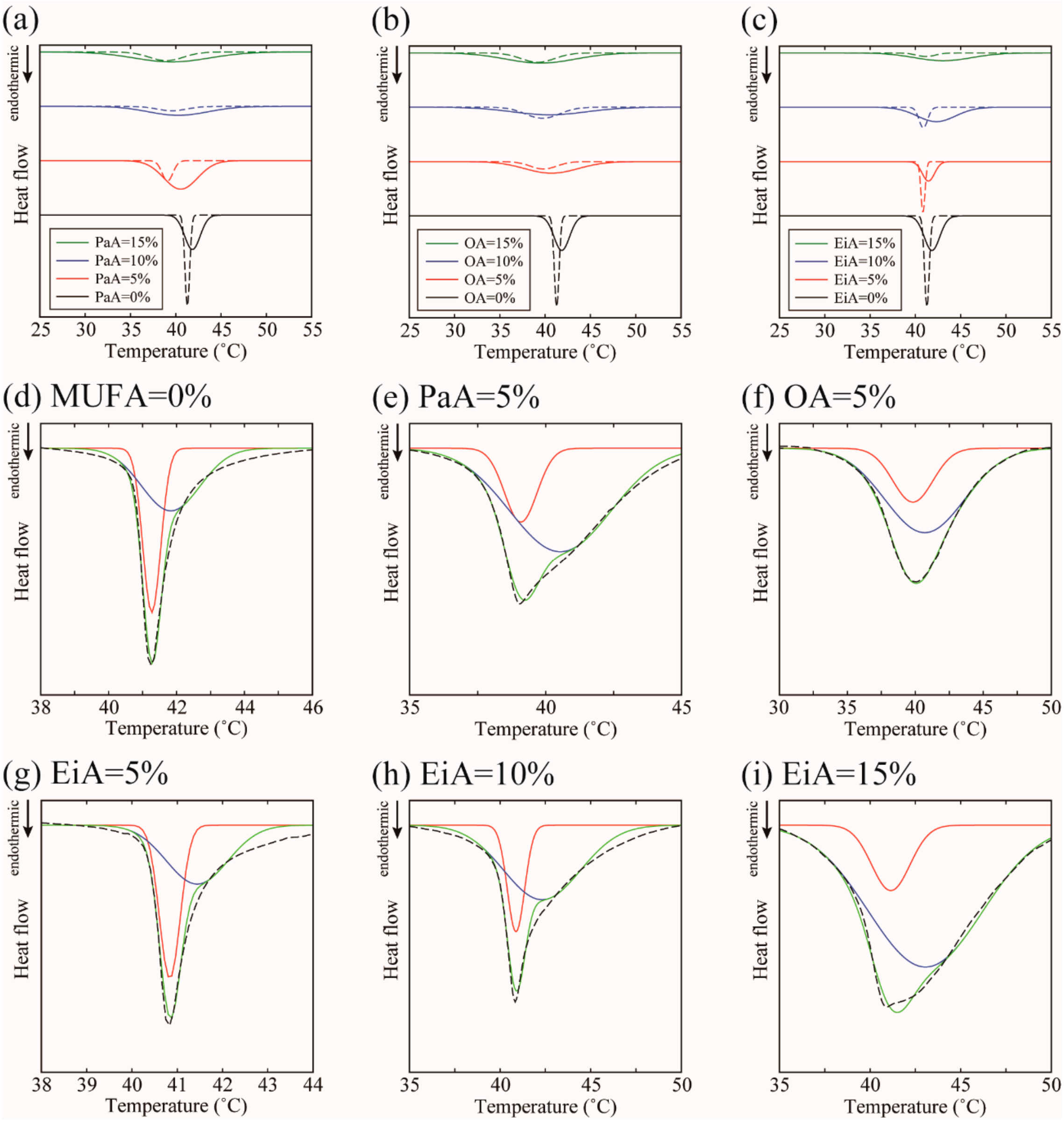

Figure S3. Peak deconvolution of DSC thermographs for (**a**) DPPC/PaA/Chol, (**b**) DPPC/OA/Chol, and (**c**) DPPC/EiA/Chol. The black, red, blue, and green lines indicate DPPC/MUFA/Chol ratios of 90/0/10, 85/5/10, 80/10/10, and 75/15/10, respectively. The solid and dashed lines indicate the higher- and lower-temperature peaks, respectively. The peak deconvolution for PaA/OA contents of 10% and 15% afforded two peak positions that were almost identical. Alternatively, the thermographs could be deconvolved into one large peak and a negligibly small peak. These results indicated that the almost symmetric thermographs obtained for these samples could be adequately fitted with a single Gaussian function, such that the assumption of two peaks used for the other samples was not valid. Therefore, the deconvolved thermographs were not used in the analysis of these samples. The deconvolved peaks with original thermographs for (**d**) DPPC/MUFA/Chol=90/0/10, (**e**) DPPC/PaA/Chol=85/5/10, (**f**) DPPC/OA/Chol=85/5/10, (**g**) DPPC/EiA/Chol=85/5/10, (**h**) DPPC/EiA/Chol=80/10/10, and (**i**) DPPC/EiA/Chol=75/15/10. The blue and red lines indicate the higher- and lower-temperature peaks, respectively. The blue and red lines correspond to the solid and dashed lines in (**a**–**c**), respectively. The light green lines are the sum of the blue and red lines and the dashed lines are the DSC thermographs shown in Figure 4 in main text. Since the plots are marked near the peaks, the scale of vertical axis is arbitrary.

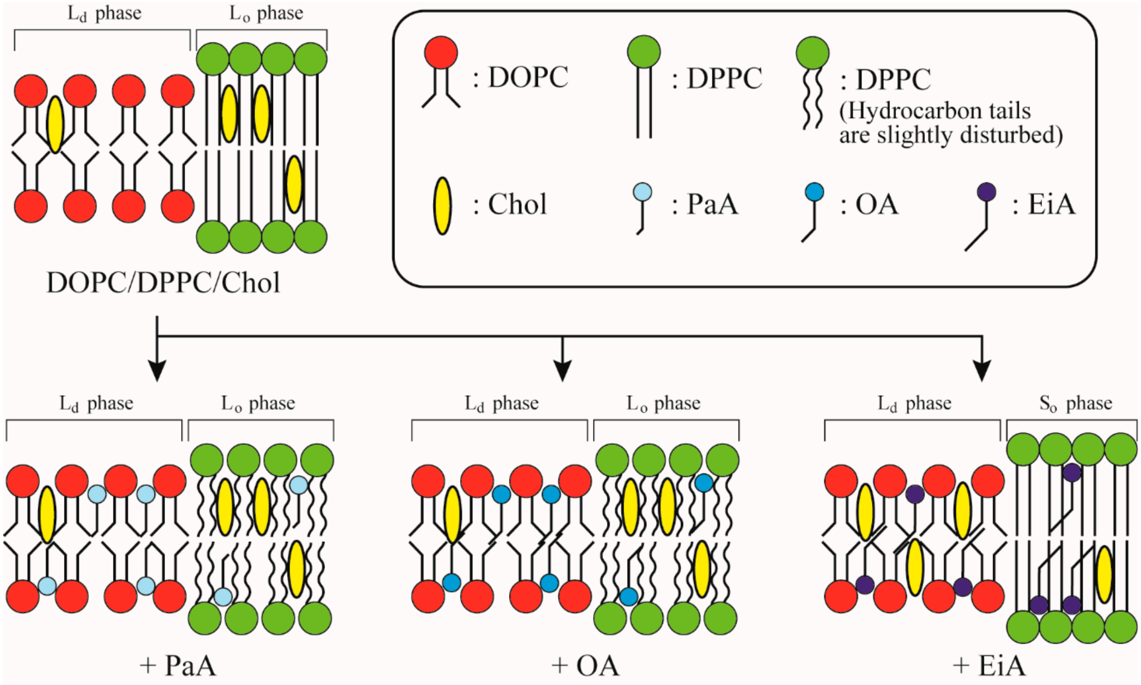

Figure S4. Schematic illustration of experimental results. The small amounts of PaA and OA are included in the L_o phase and the chain ordering of DPPC in the L_o phase is slightly disturbed. As a result, the difference of physical properties between the coexisting two phases, L_o and L_d phases, becomes smaller and the line tension at the domain boundary is reduced. On the other hand, EiA interacts more favorably with DPPC than Chol. The amount of Chol in the L_o phase is decreased and the L_o phase transforms into the S_o phase.
